# Deciphering the key pathway for triterpenoid biosynthesis in *Azadirachta indica* A. Juss.: a comprehensive review of omics studies in nature’s pharmacy

**DOI:** 10.3389/fpls.2023.1256091

**Published:** 2023-11-07

**Authors:** Nitish Dave, Atif Iqbal, Margi Patel, Tarun Kant, Virendra Kumar Yadav, Dipak Kumar Sahoo, Ashish Patel

**Affiliations:** ^1^ Genetics and Tree Improvement Division, Arid Forest Research Institute, Jodhpur, India; ^2^ Department of Life Sciences, Hemchandracharya North Gujarat University, Patan, Gujarat, India; ^3^ Department of Veterinary Clinical Sciences, College of Veterinary Medicine, Iowa State University, Ames, IA, United States

**Keywords:** terpenoid biosynthesis, transcriptomics, proteomics, genetic engineering, metabolic engineering

## Abstract

Since ancient times, *Azadirachta indica*, or Neem, has been a well-known species of plant that produces a broad range of bioactive terpenoid chemicals that are involved in a variety of biological functions. Understanding the molecular mechanisms that are responsible for the biosynthesis and control of terpenoid synthesis is majorly dependent on successfully identifying the genes that are involved in their production. This review provides an overview of the recent developments concerning the identification of genes in *A. indica* that are responsible for the production of terpenoids. Numerous candidate genes encoding enzymes that are involved in the terpenoid biosynthesis pathway have been found through the use of transcriptomic and genomic techniques. These candidate genes include those that are responsible for the precursor synthesis, cyclization, and modification of terpenoid molecules. In addition, cutting-edge omics technologies, such as metabolomics and proteomics, have helped to shed light on the intricate regulatory networks that govern terpenoid biosynthesis. These networks are responsible for the production of terpenoids. The identification and characterization of genes involved in terpenoid biosynthesis in *A. indica* presents potential opportunities for genetic engineering and metabolic engineering strategies targeted at boosting terpenoid production as well as discovering novel bioactive chemicals.

## Introduction

Neem, or *Azadirachta indica*, belongs to the family Meliaceae. It is native to India and Burma and has since been introduced to several nations in Africa and North America. *A. indica* holds significant importance in traditional medicine systems, particularly in India and other parts of South Asia (Kumar and Navaratnam, 2013; Moga et al., 2018; Blum et al., 2019).

Various biological actions have been discovered in *A. indica*, and it has been investigated for its possible antibacterial, antiviral, antifungal, molluscicidal, and antihyperglycemic characteristics (Ufele et al., 2013; Ezeigwe et al., 2015; S. Abdelhady et al., 2015; Ashfaq et al., 2016; Joy Sinha et al., 2017; Osman Mohamed Ali et al., 2017; Altayb et al., 2022).

Furthermore, the SARS-CoV-2 (COVID-19) pandemic (Kalasariya et al., 2022) has lately posed a challenge to humanity, and different compounds have been investigated in silico to treat the disease. Docking investigations of *A. indica* molecules have also provided encouraging results for their inhibitory action against various illnesses such as SARS-COV-2, malaria, and dengue (Lavanya et al., 2015; Dwivedi et al., 2016; Khanal et al., 2019; Adegbola et al., 2021; Baildya et al., 2021).

The *A. indica* has been thoroughly studied for its secondary chemical compounds and for its potential application in the discovery and synthesis of triterpenes, which are among the most abundant and highly complex families of plant-derived natural products.

A predominant focus of research in *A. indica* is the presence of an important secondary metabolite compound known as azadirachtin, which is a triterpenoid class of limonoids. Azadirachtin, the principal insecticidal component contained in the kernel of Neem seeds, displays high bioactivity against different kinds of insects (Schmutterer, 1995; Schmutterer and Singh, 1995; Hummel et al., 2015).

Growing concerns about the potential negative impacts of chemical pesticides on human health, the environment, and non-target organisms have led to an increasing preference for alternative crop protection methods (Ajiboye et al., 2022). Consequently, there is a greater focus on the development and utilization of plant- or microbe-based biopesticides that are both bioactive and biodegradable. Azadirachtin-based pesticides are environmentally friendly, biodegradable, and non-toxic to wildlife, plants, and birds (Raizada et al., 2001; Kilani-Morakchi et al., 2021). Azadirachtin has shown very minimal toxicity to mammals and has great selectivity for its target organisms (Mordue (Luntz) et al., 2005; Amaral et al., 2019). Azadirachtin is the predominant compound responsible for controlling of insects in agriculture (Vacante and Bonsignore, 2018). Over the past three decades, there has been an increase in the utilization of Neem-based insecticides, primarily attributed to the discovery and isolation of azadirachtin, the key bioactive compound responsible for its insecticidal properties (Chaudhary et al., 2017; Pasquoto-Stigliani et al., 2017). Azadirachtin has been successfully commercialized, and it is still widely accepted as being the most effective botanical pesticide that is in use in agricultural production all around the world (Isman and Grieneisen, 2014; Chaudhary et al., 2017; Aribi et al., 2020).

The biosynthesis route of Neem, which is known to synthesize physiologically and economically relevant triterpenoids with extraordinarily complex carbon skeletons and diverse functional groups, is of great interest among researchers. The first successful synthesis of azadirachtin took 20 years to complete (Jauch, 2008; Veitch et al., 2008) and comprises 71 steps; however, the yield is merely 0.00015%, and thus the production of azadirachtin at the industrial scale is not feasible.

The recent identification and functional characterization of genes involved in the formation of these triterpenoid precursors, which are responsible for the synthesize of limonoids, was made possible by studies in transcriptomics and genomics. Thus, omics research offers a useful technique for examining the biosynthesis of secondary metabolites. While the biochemical constituents of Neem have been widely investigated, its genetic, molecular, and genomic resources are scarce.

## Importance of metabolites from *Azadirachta indica*

The ability of Meliaceae plants to metabolize structurally diverse and physiologically relevant compounds is well established (Lin et al., 2022). The massive amount of literature available across several platforms makes it challenging to find information on each Neem metabolite. Azadirachtin, the most researched, has a challenging chemical structure that belongs to the tetranortriterpenoid class and is present in several forms, the most well-known of which are azadirachtin A, and azadirachtin B ((EFSA) et al., 2018; Fernandes et al., 2019).

The secondary metabolites found in various parts of the tree endow Neem with an array of biological capabilities. Azadirachtin, Azadirone, Gedunin, Nimbin, Salannin, and Vilasinin are some of the major metabolites known to exhibit substantial pesticidal and/or therapeutic properties (Dhar et al., 1998; Isman, 2006; Pravin Kumar et al., 2007; Boursier et al., 2011; Gupta and Diwan, 2017). Out of the several limonoids, azadirachtin accounts for most of its metabolite pool. **Table 1** shows the structures and properties of a few secondary metabolites.

**Table 1:** Major Secondary metabolites of *Azadirachta indica*.

Compounds	PubChem CID	MF	Structure	References
Azadirachtin	5281303	C_35_H_44_O_16_	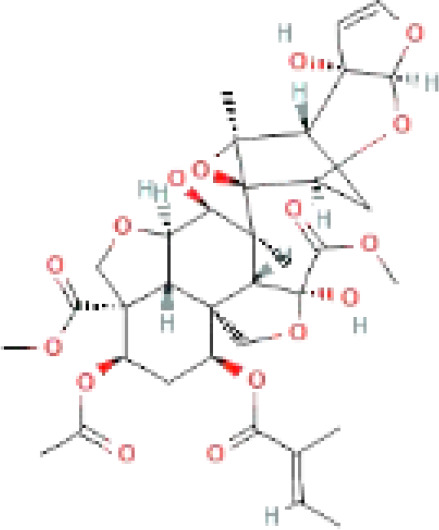	(Bartelsmeier et al., 2022)
Nimbosterol	222284	C_29_H_50_O	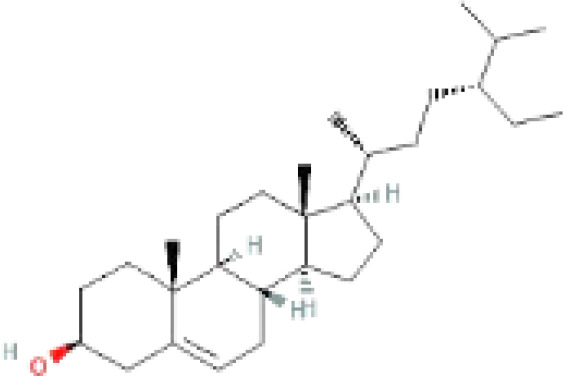	(Shrirangasami et al., 2020; Wylie and Merrell, 2022)
Nimbolide	12313376	C_27_H_30_O_7_	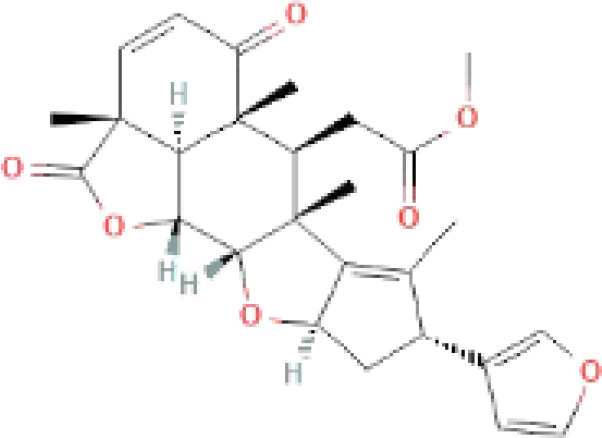	(Sarkar et al., 2021)
Palmitic acid	985	C_16_H_32_O_2_	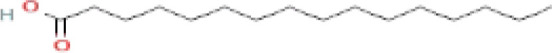	(Wylie and Merrell, 2022)
Oleic acid	445639	C_18_H_34_O_2_	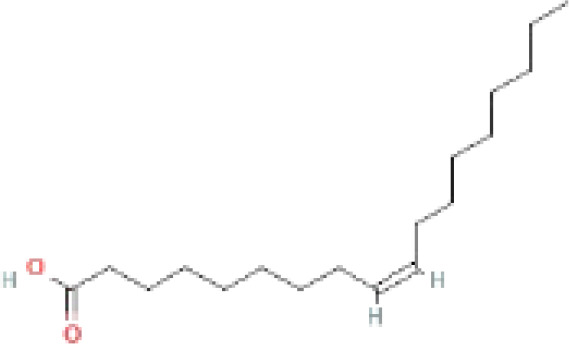	(Pasquoto-Stigliani et al., 2017)
Linoleic acid	5280450	C_18_H_32_O_2_	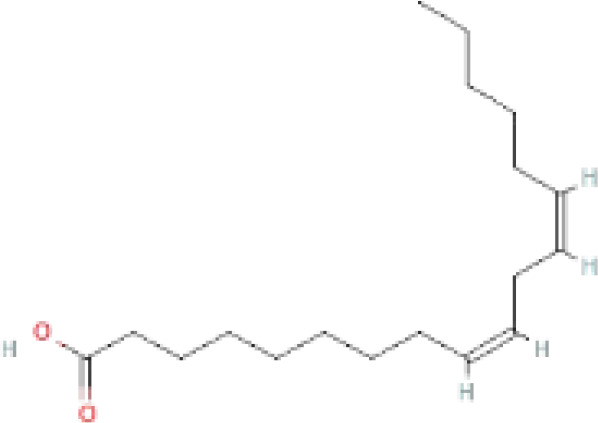	(Kaur et al., 2022)
Margocin	21632833	C_20_H_26_O_2_	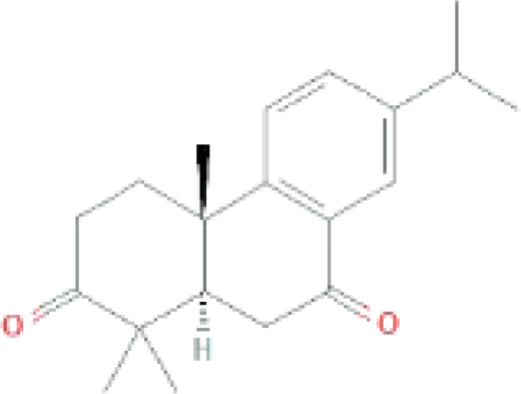	(Kumar et al., 2018; Kaur et al., 2022)
Nimbidiol	11334829	C_17_H_22_O_3_	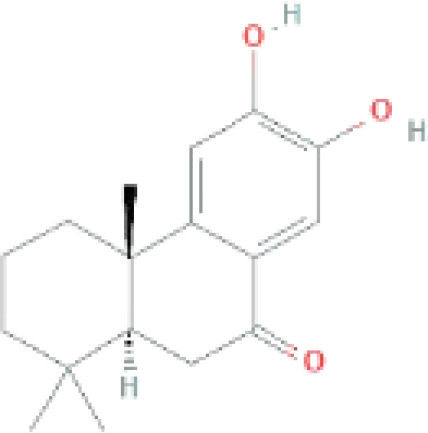	(Juin et al., 2022)
Nimbione	189404	C_18_H_22_O_3_	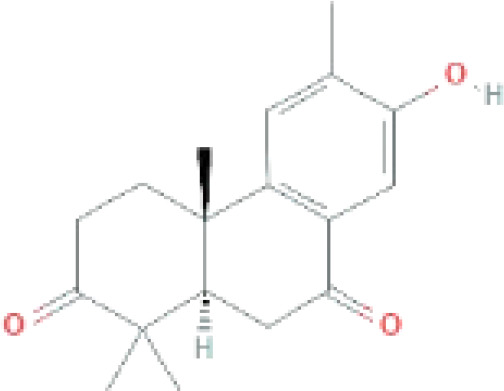	(Alzohairy, 2016)
Azadiradione	12308714	C_28_H_34_O_5_	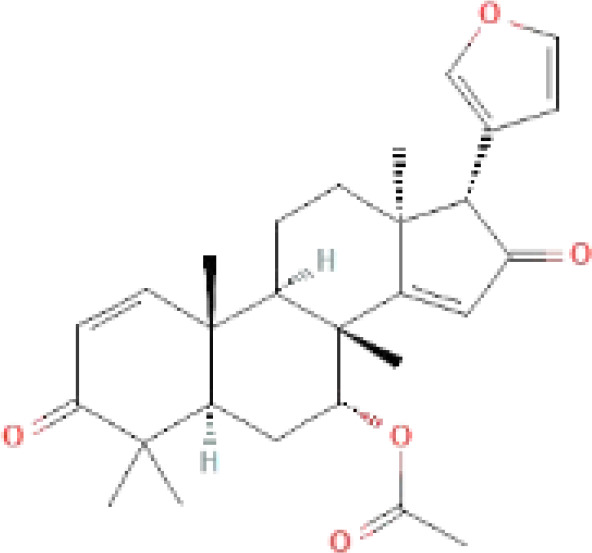	(Ponnusamy et al., 2015)
Fraxinellone	124039	C_14_H_16_O_3_	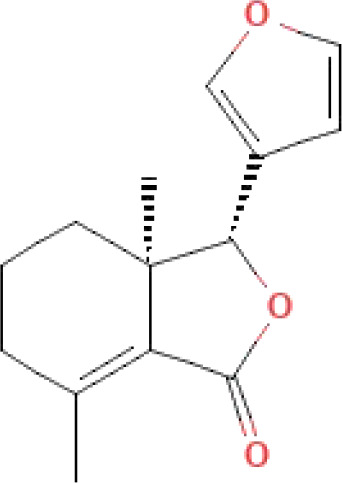	(Alzohairy, 2016; Fan et al., 2022)
Salannin	6437066	C_34_H_44_O_9_	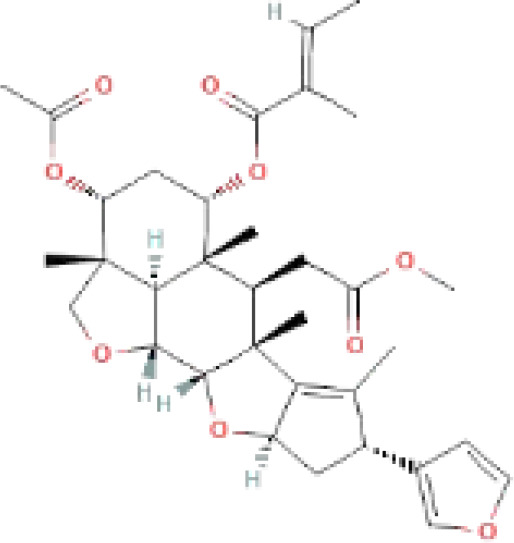	(Zhu et al., 2018)
Salannol	157144	C_32_H_44_O_8_	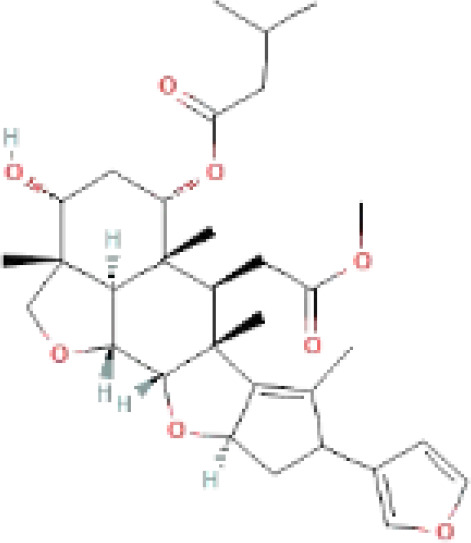	(Garg and Bhakuni, 1984; Koul et al., 2004)
Vepinin	185552	C_28_H_36_O_5_	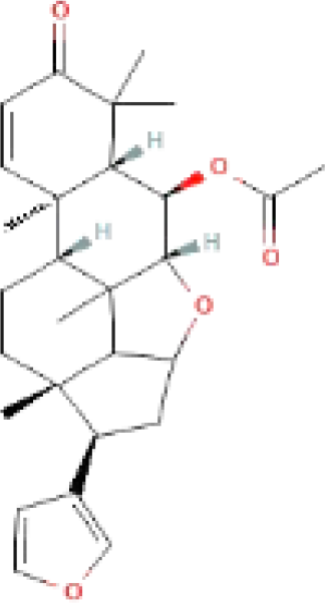	(Shrirangasami et al., 2020)
Azadirone	10906239	C_28_H_36_O_4_	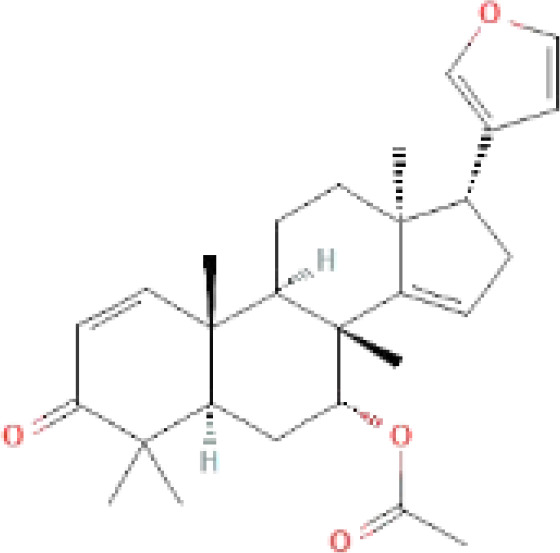	(Drijfhout and David Morgan, 2010; Akihisa et al., 2021)
Gedunin	12004512	C_28_H_34_O_7_	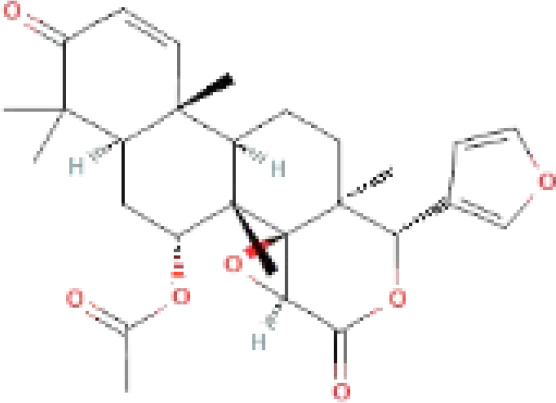	(Brandt et al., 2008)
Nimbin	108058	C_30_H_36_O_9_	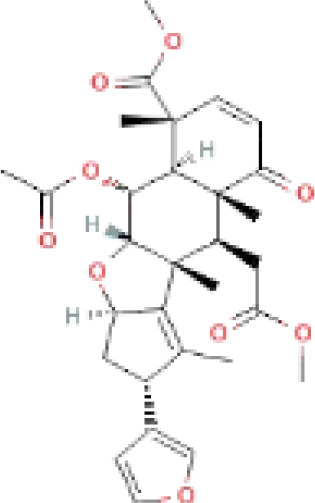	(Sarkar et al., 2022)
Desacetylgedunin	3034112	C_26_H_32_O_6_	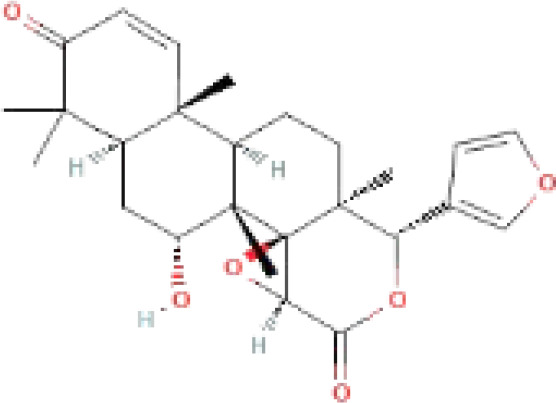	(Baildya et al., 2021)
Quercetin	5280343	C_15_H_10_O_7_	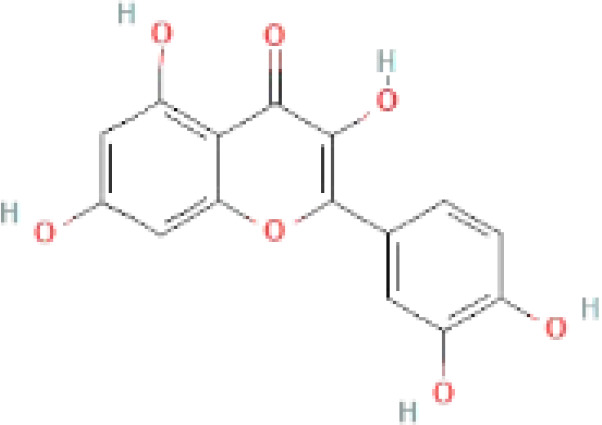	(Rao et al., 2019)

## Biosynthesis of metabolites (triterpenoids)

According to Verpoorte and Alfermann (2000), there are three main classes of secondary metabolites that may be distinguished from one another based on their biosynthetic pathways. These classes include terpenoids, polyketides, and phenylpropanoids (Verpoorte and Alfermann, 2000). Two major metabolic pathways—mevalonate (MVA) and methylerythritol 4-phosphate/deoxyxylulose 5-phosphate (MEP)—are used by plants for synthesizing terpenoids (Shi et al., 2010).

In higher plants, the traditional mevalonate pathway mostly produces the precursors, which are essential for the synthesis of sesquiterpenes, triterpenes, and sterols in the cytosol and mitochondria, while the hemi-, mono-, sesqui-, and diterpenes are produced by the non-mevalonic acid pathway.

**Figure 1:**
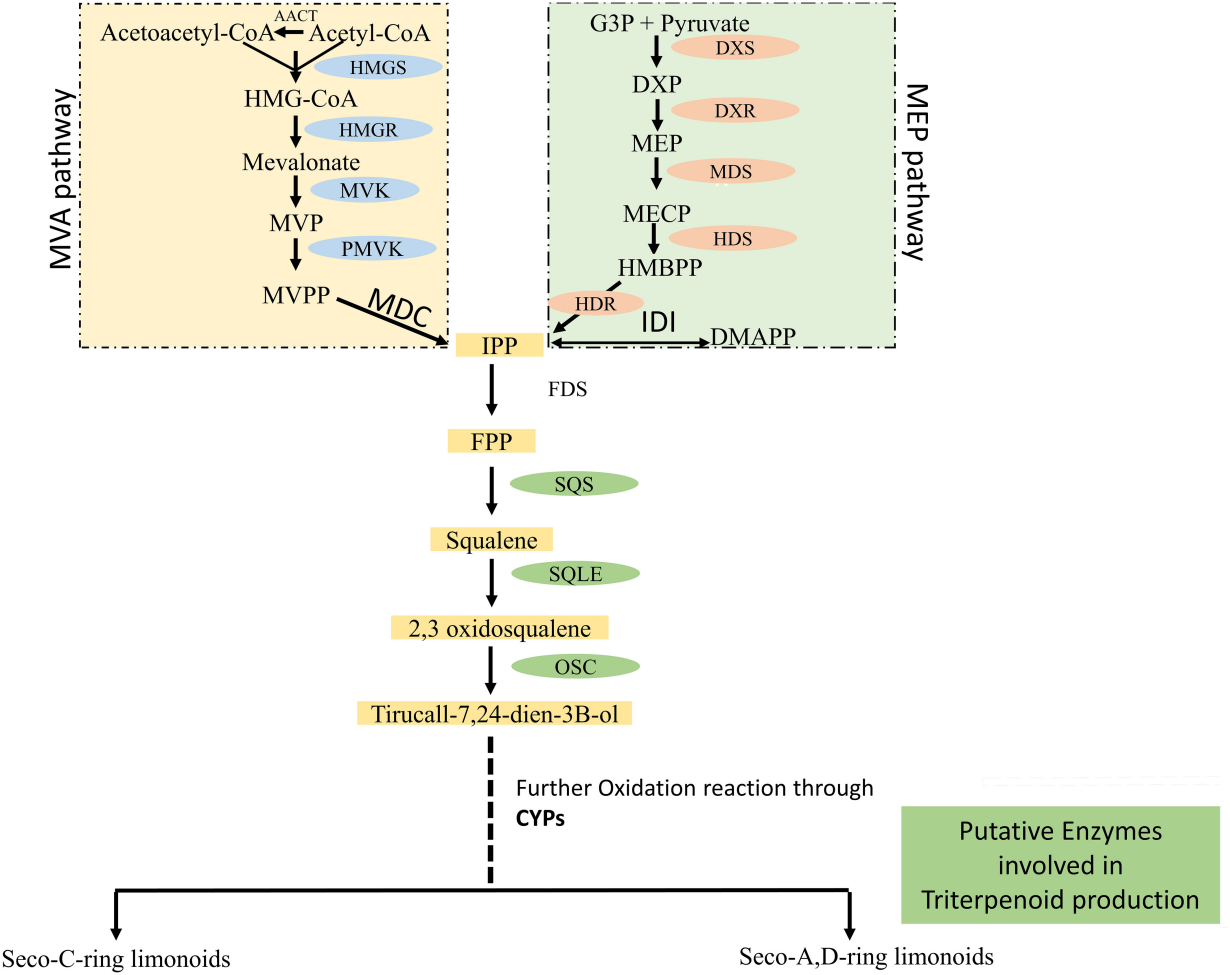
Overview of Triterpenoid Biosynthesis in *A. indica*. IPP and DMAPP are the fundamental building blocks for isoprenoid synthesis. They can combine in different ways to form larger molecules like FPP (farnesyl diphosphate). FPP can then be converted to squalene via the enzyme SQS (squalene synthase). Squalene undergoes an epoxidation reaction to form 2,3-oxidosqualene, catalyzed by the enzyme SQLE (squalene epoxidase). 2,3-oxidosqualene is then converted to different triterpenoids, such as tirucall-7,24-dien-3β-ol, through the action of the enzyme OSC (oxidosqualene cyclase). Further modifications, including oxidation reactions by various CYP (cytochrome P450) enzymes, can lead to the production of various seco-C-ring and seco-A,D-ring limonoids. (The comprehensive chemical structures for each compound have been provided in **Supplementary Table 1**).

Although the azadirachtin biosynthesis in Neem is not well established, the initial step in triterpenoid biosynthesis involves the cyclization of 2,3-oxidosqualene, which is catalyzed by oxidosqualene cyclase (**Figure 1**). This cyclization reaction represents the primary diversification level in the biosynthesis of triterpenoids (Abe et al., 1993). In addition to this, tirucallol (C30 Triterpene), a steroid of triterpenoids, is a potential precursor of Neem azadirachtin biosynthesis (Ley et al., 1993; Johnson et al., 1996; Ley et al., 2008).

In *A. indica*, two different levels of biochemical complexity are assumed to be involved in the production of azadirachtin from tirucallol (Hansen et al., 1994; Ley et al., 2008). Initially, a reduction of four atoms occurs in the lateral chain (Ley et al., 1993; Dewick, 2002), followed by the cyclization of the residual atoms to generate a furan ring. This leads to the formation of limonoids, namely azadirone and azadiradione. Following this, the C-ring undergoes an opening process, leading to the generation of C-seco-limonoids, namely nimbin, and salannin, and the third ring of apotirucallol is oxidized (Ley et al., 1993; Johnson et al., 1996; Puri, 1999; Ley et al., 2008). Additional rearrangements and oxidations are necessary to produce azadirachtin, which is classified as one of the most extensively oxidized triterpenoids (Aerts and Mordue, 1997).

## Identification of candidate genes responsible for triterpenoid synthesis in *A. indica* through genomics

### Genome studies in *A. indica*

Genomic studies in Neem have focused on sequencing and analyzing the complete set of genes and genomic elements present in the species. The first draught genome was published by a team led by Krishnan (Krishnan et al., 2012). The investigators reported diverse insights from the genome of *A. indica*. The researchers identified genes such as Terpene Synthase 21 (*TPS21)*, 4-hydroxy-3-methylbut-2-enyl diphosphate reductase (*lytB)*, 4-hydroxy-3-methylbut-2-en-1-yl diphosphate reductase (*ispH)*, 4-diphosphocytidyl-2-C-methyl-D-erythritol kinase (*ispE)*, Geranylgeranyl diphosphate synthase (*GGPS)*, Farnesyl diphosphate synthase (*FDPS*), squalene synthase (*FDFT1)*, and Squalene epoxidase (*SQLE)* that are involved in terpenoid production and are also associated with steroid biosynthesis pathways. These genes were observed to be more abundant in Neem compared to *Arabidopsis thaliana*, *Oryza sativa*, *Citrus sinensis*, and *Vitis vinifera*. According to their report, it was found that the genome of *A. indica* is characterized by a high AT content, a low abundance of repetitive DNA sequences, and a mean gene length of 1.69Kb. Additionally, *A. indica* was observed to be phylogenetically related to *Citrus sinensis* (Krishnan et al., 2012).

However, in another study, the genome published by Kuravadi and their group reported the presence of about 87 megabases (Mb) of repetitive DNA sequences in the Neem genome, accounting for approximately 33% of the total genome size. This percentage is higher than what was previously reported, suggesting a significant presence of repetitive elements in the Neem genome. Furthermore, the study identified molecular markers such as SSRs (Simple Sequence Repeats), SNPs (Single Nucleotide Polymorphisms), and InDels (insertions and deletions) within the Neem genome. These markers can serve as genetic signposts, allowing researchers to identify and study elite Neem genotypes with desirable traits. The genome was also compared with the citrus genome, which revealed extensive syntenic blocks between Neem and citrus chromosomes, indicating genetic relatedness (Kuravadi et al., 2015).

A recent study conducted by Du and their groups successfully reported the genome of *A. indica* at a chromosome-scale level. The assembled genome had a size of approximately 281Mb, covering around 73.2% of the estimated total genome size. This achievement of chromosome-scale assembly provides a comprehensive understanding of the Neem genome. Moreover, they reported that the Neem genome exhibited a high level of heterozygosity (0.896%), indicating significant genetic diversity within the species. They also reported that *A. indica* possesses a higher number of terpene-related gene clusters compared to other species, and chromosome 13 played a central role in the evolution of terpenoid biosynthesis in *A. indica*. They observed that a lot of genes linked to terpenes were clustered on this chromosome. This shows that chromosome 13 may have gone through certain types of evolution that led to the accumulation and organization of genes related to terpenes in the Neem genome (Du et al., 2022).

According to Du and their team, there are 70 terpene synthase (*TPS*) genes and 355 cytochrome P450 (*CYP*) genes that were responsible for terpenoid biosynthesis. The abundance of TPS genes in *A. indica* was consistent with other terpenoid-rich plant species. Notably, the *A. indica TPS* and *CYP* genes were mostly found in the terpene-related groups on chromosome 13, which further suggests that they might be involved in the biosynthesis of azadirachtin (Du et al., 2022). **Table 2** shows a comparison of the different genomes of *A. Indica*.

**Table 2:** Comparison of the *A. indica* genomes.

	(Du et al., 2022)	(Kuravadi et al., 2015)	(Krishnan et al., 2012)
Sequence technology	Illumina + PacBio + Hi-C	Illumina	Illumina + PacBio
Assembly	Chromosome	Contig	Scaffold
**Genome** size (Mb)	281	261.2	216
GC%	32.2	32	31.9
no. of Scaffold	70	126,142	25,560
Scaffold n50 (bp)	1,95,42,739	3,491	26,29,187
Number of contigs	870	142,701	48,555
Contig n50 (bp)	60,39,544	3,310	25,406
BUSCO	91.70%	79.90%	91.40%

### Transcriptomic studies in *A. indica*: genes involved in azadirachtin biosynthesis

**Figure 2:**
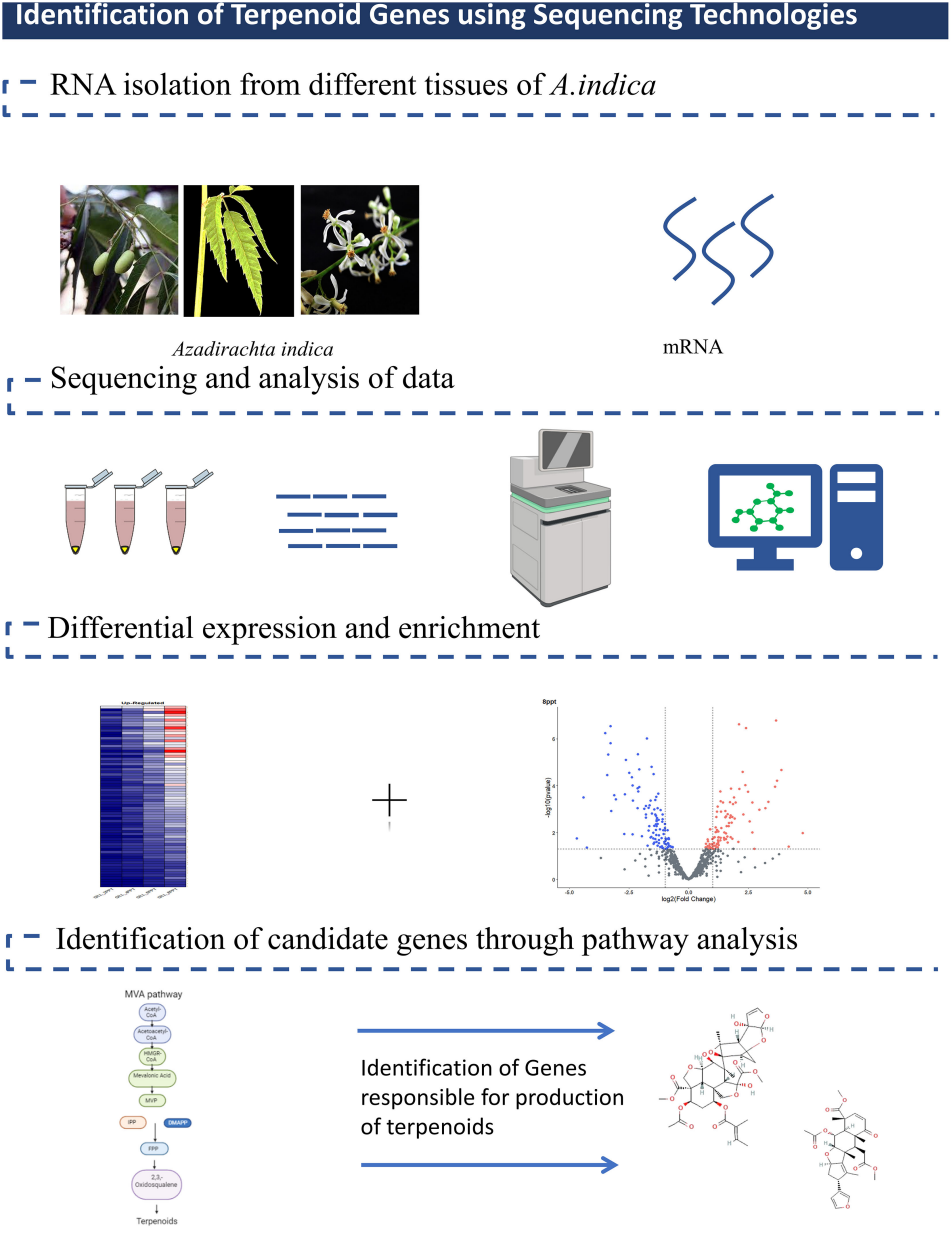
Identification of genes involved in Terpenoid Biosynthesis.

Transcriptomic studies have been performed to examine gene expression patterns and identify differentially expressed genes in various tissues and under different conditions (**Figure 2**). RNA-seq technology has been utilized to analyze the transcriptomes of *A. indica* leaves, flowers, seeds, and other tissues, providing valuable information on gene expression dynamics and regulatory networks.

Krishnan and their group published the first draught genome and Transcriptome from various parts of *A. indica*. They conducted phylogenetic studies that confirmed the taxonomic closeness between Neem and citrus, which also belong to the same order. Also, Neem was found to be related to Melia species, which is another plant that has terpenoid chemicals. This suggests that these chemicals are made in the Meliaceae family by a similar evolutionary process (Krishnan et al., 2012; Krishnan et al., 2016).

The tissue-specific variation was also identified in triterpenoids (Pandreka et al., 2015). Their findings indicated that the mature seed kernel and pericarp of *A. indica* during the early stages contained the highest levels of triterpenoids. Furthermore, as compared to other tissues, the kernel contained a diverse range of triterpenoids, particularly C-seco triterpenoids. They identified and functionally characterized the genes which are involved in the initial steps of isoprenoid biosynthesis, such as *AiGDS*, *AiFDS*, and *AiSQS*. They also examined the levels of 15 triterpenoids in various Neem tissues, including flowers, leaves, stem, bark, and different developmental stages of pericarp and kernel. Using solvent partition for extraction and UPLC-ESI(+)-HRMS for analysis, they observed that the concentration of these triterpenoids varied among the tissues. Notably, kernel displayed the highest triterpenoid content. This experimental aspect was aimed at corelates with the omics data with actual triterpenoid levels in various tissues. In another study conducted by (Bhambhani et al., 2017), various developmental stages of the fruit (FS1, FS2, FS3, FS4) and leaves of *A. indica* were sampled from a five-year-old tree. Upon conducting a phytochemical analysis focused on tetranortriterpenoids, several observations were made. Azadirachtin displayed a fruit-specific accumulation, reaching its peak in the FS3 stage. While nimbin was present in the leaves, it accumulated more significantly in the fruit stages. Notably, both azadirachtin and salannin were absent in the leaf tissue. Furthermore, only a trace amount of nimbin was found in the leaves, underscoring the observation that the fruit, particularly the FS3 stage, is richer in these phytochemicals compared to the leaves (Bhambhani et al., 2017).

An important enzyme was functionally characterized in a significant study by a team led by Hodgson. The researchers characterized the tirucalla-7,24-dien-3β-ol synthase, which is an Oxidosqualene Cyclase (*OSCs*), from three distinct plant species: *A. indica, Melia azedarach*, and *Citrus sinensis*. They also identified three cytochrome P450 (*CYP*) sequences, namely *AiCYP71BQ5*, *AiCYP72A721*, and *AiCYP88A108*, which showed high co-expression with *AiOSC1*. The study suggested that certain Cytochrome P450 enzymes (*CYPs*) could potentially be responsible for oxidizing the tirucalla-7,24-dien-3β-ol scaffold generated by *AiOSC1*. Furthermore, it was observed that *AiOSC1* showed the highest expression in the fruit, aligning with a previous report that highlighted elevated levels of ring-intact limonoids, such as azadiradione and epoxyazadiradione, in the fruit of *A. indica* (Hodgson et al., 2019). A Group led by Pandreka, also cloned and functionally characterized tirucalla-7,24-dien-3β-ol synthase (*AiTTS1*), an enzyme responsible for the synthesis of tirucalla-7,24-dien-3β-ol. Additionally, they cloned and characterized squalene epoxidase (*AiSQE1*), cycloartenol synthase (*AiCAS*), and two cytochrome P450 reductases. Through comparative tissue expression analysis, the researchers also identified genes involved in terpenoid synthesis and found higher levels of expression for *AiFDS* (farnesyl diphosphate synthase), *AiSQS* (squalene synthase), *AiSQE3* (squalene epoxidases), and *AiTTS1* (triterpene synthases) in the kernel (Pandreka et al., 2021).

In another study, Wang et al. (2020) used a novel hybrid-sequencing approach using Illumina HiSeq and Pacific Biosciences, and they identified five different types of genes potentially involved in azadirachtin biosynthesis. They identified 22 unigenes encoding enzymes, including the oxidosqualene cyclase (OSC), alcohol dehydrogenase (ADH), cytochrome P450 (CYP450), acyltransferase (ACT), and esterase (EST). **Table 3** shows the comparisons of different attempts taken by various researchers (Wang et al., 2020).

**Table 3:** Detailed comparison of transcriptomes by various authors.

Tissue/Condition	Methodology/Sequencing Platform	Assembly approach	Annotation approach	Author/References
**Root, Leaf, Stem, and Flower**	WGS and RNA Seq Solexa sequencing-by-synthesis	SOAPdenovo, Trinity	BLAST2GO, GlimmerHMM, PASA, KEGG	(Krishnan et al., 2012; Krishnan et al., 2016)
**Fruit, flower, and leaf**	RNA-seq	Velvet	BlastX, and KEGG	(Pandreka et al., 2015)
**Flower and bud, fruit coat and pulp, developing endosperm, mature fruit, seedling root, drought root, drought shoot, albino root, albino shoot, leaf callus**	WGS and RNA-seq	Velvet, 454 reads were assembled using MIRA	BlastX, GO, KEGG, Enzyme Commission	(Kuravadi et al., 2015)
**Adventitious root and leaf**	RNA-seq	Trinity	TAIR and NCBI NR database, GO Annotation	(Wang et al., 2016)
**Mature leaf (ML) and fruit**	RNA-seq	–	BlastX, TAIR	(Bhambhani et al., 2017)
**Root, leaf, stem, flower, and fruit containing seed**	RNA-seq	Trinity	BlastX, Swiss Prot, COG, KEGG, HMMER 3.0	(Wang et al., 2020)
**Kernel, pericarp, leaves, and flower**	RNA-seq	Trinity	Blastx, and KEGG	(Pandreka et al., 2021)
**Leaves**	WGS	Canu, RACON, Pilon, ALLHIC, HiC-Pro	NR, InterPro, Swiss-Prot, EggNOG	(Du et al., 2022)

## Discussion

Omics is a potent tool for identifying essential genes for significant traits, clarifying physiological event mechanisms, and revealing unknown metabolic pathways. A whole genome sequence provides a complete overview of how the functional elements of the genome are structurally organized. These structural elements carry the knowledge of the evolutionary history of an organism (Subramanian et al., 2020).

In addition to genomes, transcriptomes have also been shown to be essential in deciphering the molecular mechanisms and metabolic pathways underpinning a wide range of biological functions. High-throughput sequencing technologies such as RNA sequencing (RNA-seq) are utilized to create extensive transcriptome atlases, and these technologies also contribute to a better knowledge of the functional components that make up the genome of any species (Jiang et al., 2015).

Considering the advantages of omics technology can aid in identifying the unexplored pathways across different species, and the integration of transcriptome data with other omics approaches, such as proteomics and metabolomics, can provide a more comprehensive understanding of the biology of any given species (Yan et al., 2022). By correlating gene expression with protein abundance and metabolite levels, researchers can unravel the complex interactions and regulatory networks underlying physiology and biochemistry.

In the case of *A. indica*, the biosynthetic pathway for triterpenoid production was not well studied until the publication of the first genome and transcriptome of *A. indica*. These studies helped in identifying the repeat elements, the nucleotide composition of nucleotides, and expression profiles of initial genes involved in terpenoid production in different tissues of Neem. *A. indica* was the first Meliaceae family member to be sequenced genome-wide (Krishnan et al., 2012).

The Relative expression of *HMGR* (HMG-CoA reductase) was higher when compared to the MEP pathway, confirming that the Mevalonate pathway might contributes to the isoprene units of triterpenoids. The distribution of limonoids varies across different tissues reported (Pandreka et al., 2015; Aarthy et al., 2018).

Some of the critical enzyme like oxidosqualene cyclase (*AiOSC1*) involved in the pathway for the synthesis of triterpenoid was reported by (Hodgson et al., 2019), but they failed to mention triterpene synthase (*TTSs*), including tirucalla-7, 24-dien-3β-ol synthase, which were functionally characterized by different researchers (Pandreka, 2018; Thulasiram et al., 2018). These Triterpene Synthases (*TTSs*) work on 2,3-oxidosqualene to create cyclic compounds, which is the crucial step for steroid and triterpenoid biosynthesis (Volkman, 2005). Later, Triterpene synthase (*TTS1*), was also functionally characterized by a team led by Pandreka (Pandreka et al., 2021).

These clusters of genes mentioned by the various authors from 2012 to 2022 have paved the way for the identification of genes responsible for the production of industrially and medically important triterpenoids in *A. indica*. The integration of omics, bioinformatics, and genetic engineering technologies holds a great deal of promise for expanding our understanding of the process and locating any missing links in the chain of events that led to the creation of azadirachtin (Kumar et al., 2021). Although there aren’t any well-established genes involved in the numerous processes leading from tirucallol to azadirachtin, utilizing these methodologies can nevertheless yield really helpful insights.

Researchers are able to conduct an exhaustive investigation of the genetic and molecular components that are associated with a pathway by making use of omics tools such as genomics, transcriptomics, and proteomics. Tools and techniques from the field of bioinformatics can be used to assist in the processing and interpretation of huge amounts of biological data, which can in turn facilitate the discovery of candidate genes and probable enzymes involved in this class of triterpenoids.

The discovery of the missing link in the biosynthesis of azadirachtin in *A. indica* presents a significant difficulty because the biosynthesis of azadirachtin involves a complicated network of processes, with metabolites serving as both substrates and products and gene products acting as enzymes that catalyze the appropriate reactions. Once the underlying molecular mechanisms are understood, new opportunities arise for altering and optimizing the production of azadirachtin, which may result in increased yields or novel applications in the pharma and agriculture industries.

## Conclusion and future perspectives

*A. indica* is classified as a high-value medicinal tree and a great source for azadirachtin, which is known for its insecticidal activity. Along with these molecules, *A. indica* also synthesizes several other biologically active compounds with demonstrated pharmacological attributes. Despite extensive research on *A. indica’s* enzymatic pathway machinery, the downstream genes responsible for azadirachtin production have not yet been identified. This knowledge gap highlights the need for in-depth studies that employ an integrated omics approach. By combining various omics technologies, such as genomics, transcriptomics, proteomics, and metabolomics, researchers can gain a comprehensive understanding of the biosynthesis of different alkaloids and their analogs present in *A. indica*. Identifying and isolating these compounds is crucial for their industrial and pharmacological applications. With the help of omics tools, the biosynthesis pathways of several bioactive substances in *A. indica* can be mapped out and can provide more useful genetic information about *A. indica*. The potential of omics research in *A. indica* appears to be promising, presenting prospects for enhancing our understanding of Neem’s biosynthesis pathways, identifying new bioactive compounds, evaluating genetic variability, expediting functional gene discovery, directing breeding endeavors, and implementing systems biology methodologies. These advancements can have significant implications for the development of Neem-based applications in the medical, agricultural, and industrial sectors.

## Author contributions

ND: Data curation, Formal analysis, Investigation, Methodology, Validation, Visualization, Writing- original draft. AI: Data curation, Formal analysis, Investigation, Methodology, Validation, Visualization, Writing- original draft. MP: Data curation, Formal analysis, Investigation, Methodology, Software, Writing- review & editing. TK: Conceptualization, Funding acquisition, Project administration, Resources, Software, Supervision, Writing- original draft. VY: Conceptualization, Project administration, Supervision, Visualization, Writing- original draft, Writing- review & editing. DS: Conceptualization, Funding acquisition, Project administration, Resources, Software, Supervision, Writing- original draft. AP: Conceptualization, Project administration, Supervision, Visualization, Writing- original draft, Writing- review & editing.
