# Supplementary material for: Deciphering the key pathway for triterpenoid biosynthesis in Azadirachta indica A. Juss.: a comprehensive review of omics studies in nature’s pharmacy
Source: Front Plant Sci. 2023 Nov 7;14:1256091. doi: 10.3389/fpls.2023.1256091 (PMC10664250; doi:10.3389/fpls.2023.1256091)
Supplement: Supplementary file 1 [file Table_1.docx]

**SUPPLEMENTARY TABLE 1** Overview of key chemical entities, focusing on their abbreviations, nomenclature, associated PubChem CID, and molecular structures.

| **Abbreviation** | **Full Name** | **PubChem CID** | **Chemical Structure Depiction** |
| --- | --- | --- | --- |
| Acetyl-CoA | Acetyl coenzyme A | 444493 | 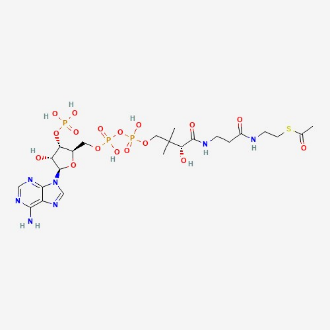 |
| Acetoacetyl-CoA | Acetoacetyl coenzyme A | 92153 | 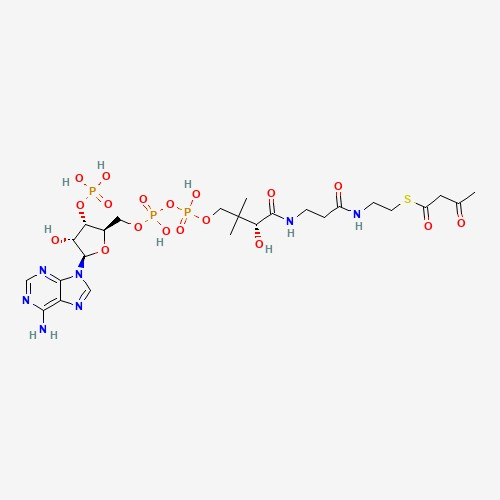 |
| HMG-CoA | 3-Hydroxy-3-methylglutaryl coenzyme A | 15983953 | 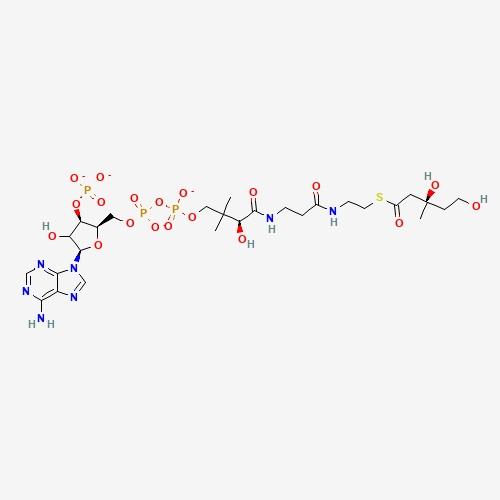 |
| Mevalonate | (R)-Mevalonic acid | 5288798 | 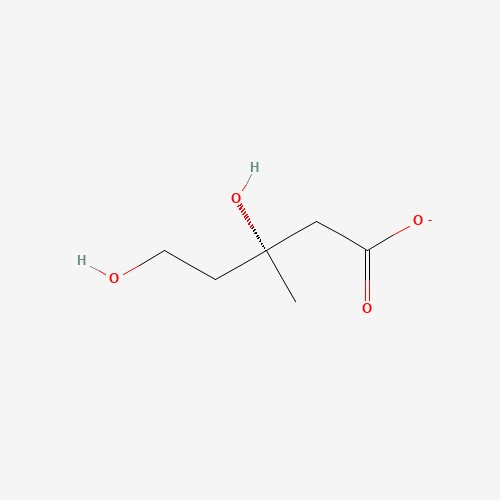 |
| MVP | Mevalonate-5-Phosphate | 439400 | 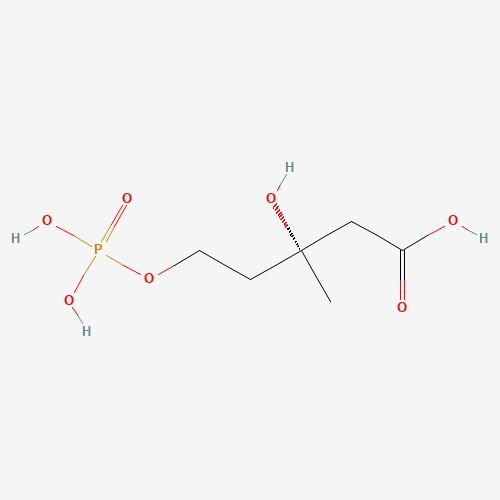 |
| MVPP | Mevalonate-5-Diphosphate | 439418 | 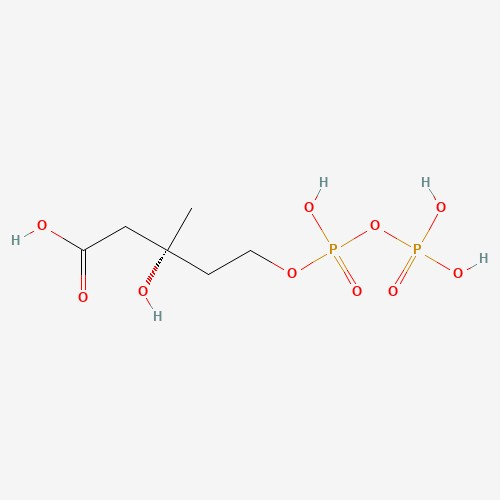 |
| G3P | Glyceraldehyde 3-phosphate | 439168 | 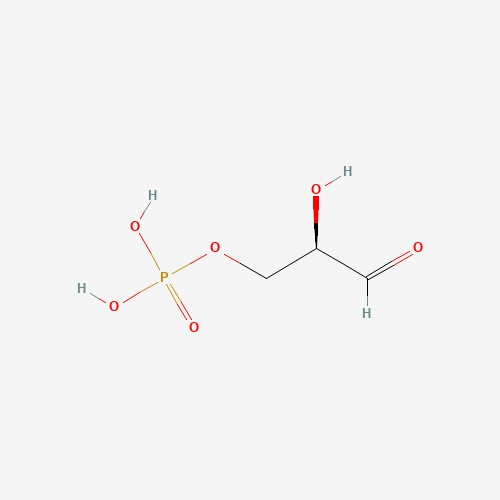 |
| Pyruvate | Pyruvic acid | 1060 | 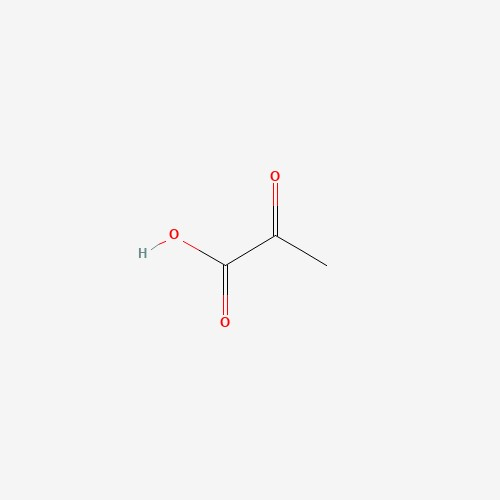 |
| DXP | 1-Deoxy-D-xylulose 5-phosphate | 443201 | 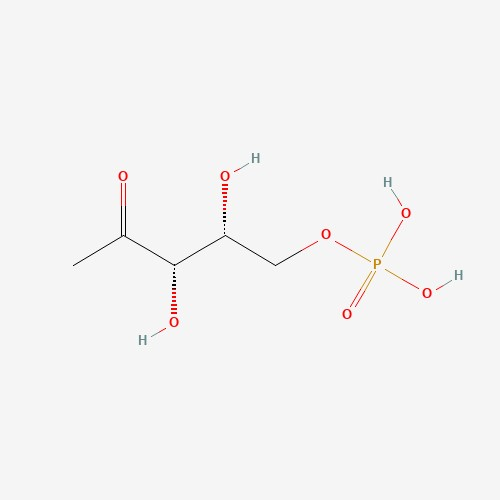 |
| MEP | Methyl-D-erythritol 4-phosphate | 443198 | 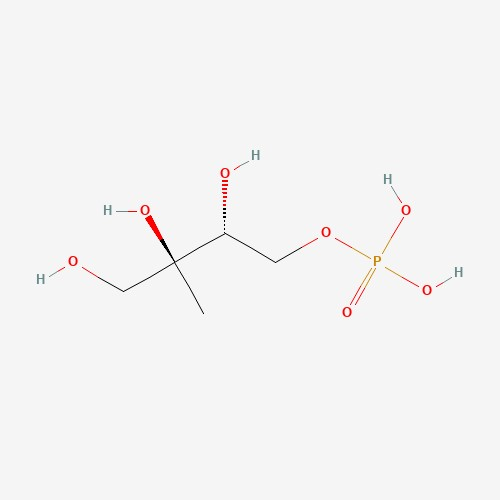 |
| MECP | 2C-METHYL-D-ERYTHRITOL 2,4-CYCLODIPHOSPHATE | 126747 | 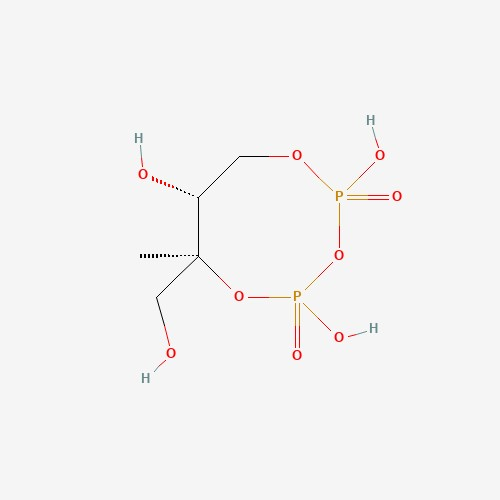 |
| DMAPP | Dimethylallyl pyrophosphate | 647 | 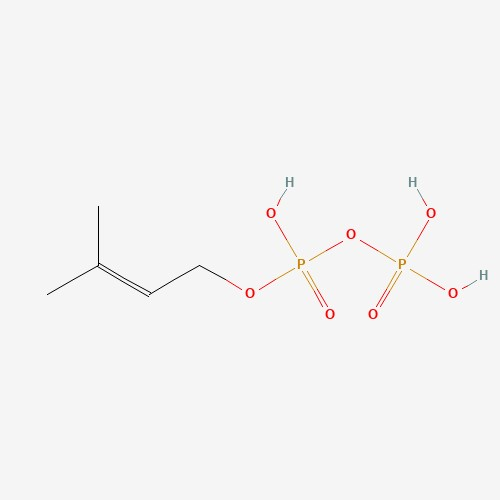 |
| IPP | Isopentenyl pyrophosphate | 1195 | 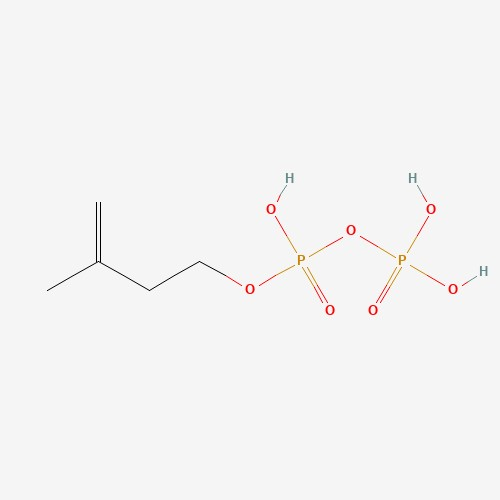 |
| FPP | Farnesyl pyrophosphate | 445713 | 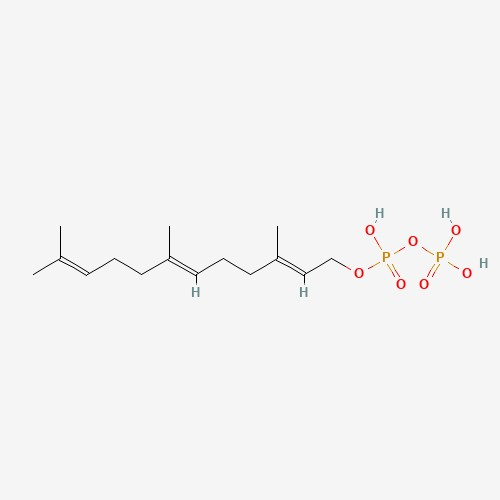 |
| SQLE | Squalene | 638072 | 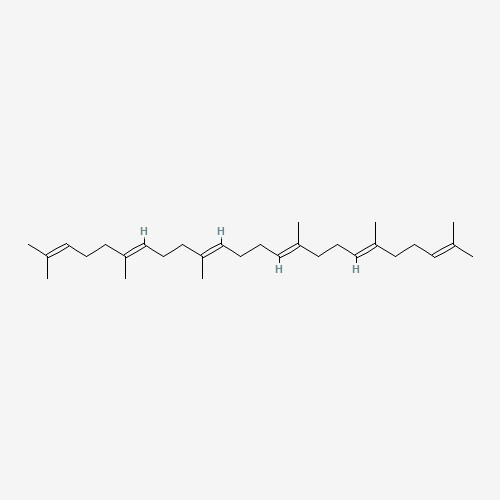 |
| 2,3 OSC | 2,3 oxidosqualene (squalene with an epoxide group at positions 2 and 3) | 5366020 | 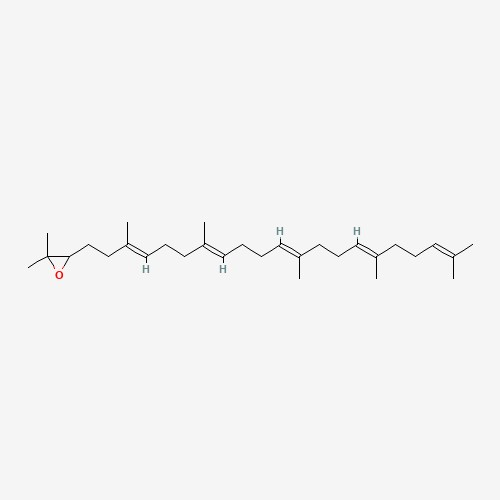 |
| Tirucallol | Tirucall-7,24-dien-3B-ol | 101257 | 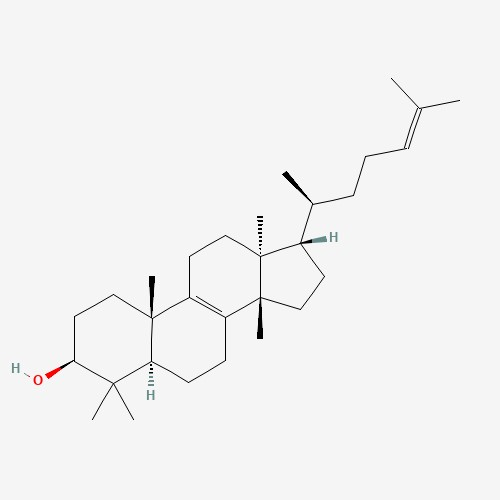 |
